# Supplementary material for: Biomimetic single Al-OH site with high acetylcholinesterase-like activity and self-defense ability for neuroprotection
Source: Nat Commun. 2023 Sep 28;14:6064. doi: 10.1038/s41467-023-41765-x (PMC10539540; doi:10.1038/s41467-023-41765-x)
Supplement: Supplementary file 1 — Supplementary Information [file 41467_2023_41765_MOESM1_ESM.pdf]

Supplementary Information

Biomimetic Single Al-OH Site with High Acetylcholinesterase-Like Activity and Self-Defense Ability for Neuroprotection

Weiqing Xu[1], Xiaoli Cai[2], Yu Wu[1], Yating Wen[1], Rina Su[1], Yu Zhang[1], Yuteng Huang[2], Qihui Zheng[2], Liuyong Hu[3], Xiaowen Cui[4], Lirong Zheng[1], Shipeng Zhang[5], Wenling Gu[1], Weiyu Song[6], Shaojun Guo[5]✉, Chengzhou Zhu[1]✉

[1]National Key Laboratory of Green Pesticide, International Joint Research Center for Intelligent Biosensing Technology and Health, College of Chemistry, Central China Normal University, Wuhan 430079, P.R. China.

[2]Department of Nutrition, Hygiene and Toxicology, School of Public Health, Medical College, Wuhan University of Science and Technology, Wuhan 430065, P.R. China.

[3]School of Materials Science and Engineering, Wuhan Institute of Technology, Wuhan 430205, P.R. China.

[4]Beijing Synchrotron Radiation Facility, Institute of High Energy Physics Department, Chinese Academy of Sciences Institution, Beijing 100049, P.R. China.

[5]School of Materials Science and Engineering, Peking University, Beijing 100871, P.R. China

[6]State Key Laboratory of Heavy Oil Processing, College of New Energy and Materials, China University of Petroleum, Beijing 102249, P.R. China

✉Email: czzhu@ccnu.edu.cn; guosj@pku.edu.cn

Experimental

Materials. Zirconyl chloride octahydrate ($ZrOCl_2·8H_2O$), 1,4-dicarboxybenzene, aluminum nitrate nonahydrate ($Al(NO_3)_3•9H_2O$), N-ethylmorpholine, 5,5'-dithiobis-(2-nitrobenzoic acid) (DTNB), N-methylacridone (NMA), riboflavin, and nitroblue tetrazolium (NBT) were purchased from Shanghai Aladdin Bio-Chem Technology Co., Ltd. Acetylcholinesterase (AChE), acetylcholine chloride (ACh), thioacetylcholine chloride (ATCh), choline, choline oxidase (ChOx), and Al_2O_3 nanomaterials were from Sigma-Aldrich. Diethyl-4-nitrophenylphosphate (DENP) was purchased from Putian Tongchuang Technology Co., Ltd. 3,3',5,5'-tetramethylbenzidine (TMB) was obtained from Shanghai Dibai Biotechnology Co., Ltd. was from Shanghai Medpep Co., Ltd. All cell culture and assay reagents were purchased from. Elabscience Biotechnology Co., Ltd. Other chemical reagents were purchased from Sinopharm Chemical Reagent Co., Let (Shanghai, China).

Instruments. Transmission electron microscopy (TEM) studies were performed by using a FEI Titan G2 60-300 (super-x). Scanning electron microscopy (SEM) images were obtained from the Gemini SEM 300 (ZEISS). X-ray absorption near-edge structure (XANES) and extended X-ray absorption fine structure (EXAFS) experiments on a Zr K-edge were obtained from 1W1B station in Beijing Synchrotron Radiation Facility (BSRF). The element contents were obtained by inductively coupled plasma optical emission (ICP-OES) spectrometry (Agilent 8800). Nitrogen sorption measurements were conducted using a fully automatic specific surface and aperture analyzer (Quantachrome, USA). Powder X-ray diffraction (XRD) patterns were carried out using a Tensor 27. X-ray photoelectron spectroscopy (XPS) measurements were used by a VG Multilab 2000 (Thermo Fisher, USA). Electron paramagnetic resonance (EPR) measurements were obtained by an EMXmicro-6/1 (Bruker, Germany). In-situ ATR-FTIR

analysis was performed using a Nicolet iS50 FTIR spectrometer (Thermo) equipped with a diamond internal reflection element (IRE) (refractive index diamond n = 2.4, incidence angle r = 42°). Ultrapure water was obtained from a Milli-Q purification system (Millipore, MA, USA). Confocal laser scanning microscopy (CLSM) experiment utilized a Leica TCS SP8 microscope. All the absorbance and fluorescence spectra were obtained from a multimode reader (Tecan Spark, Switzerland).

Synthesis of MOF-808. First, $ZrOCl_2 \cdot 8H_2O$ (970 mg, 3 mmol) was dissolved in formic acid (30 mL) and added into the 1,3,5-benzenetricarboxylic acid solution (210 mg, 1.26 mmol) in 30 ml DMF. The mixed solution was then treated by ultrasound for about 10 mins before being transferred to a Teflon-capped glass jar. After the reaction at 110 °C for 24 h, the solid was obtained by centrifuging (9500 xg, 3 min) and washing with DMF and acetone three times. Finally, through a solvent exchange process in the acetone for 2 days, MOF-808 was prepared by vacuum drying at 100 °C.

Quantification of Lewis acidity by NBT colorimetric method. Both NBT solution (0.1 mM) and MOFs suspensions were first degassed with N_2 for 30 mins. The MOFs (10 μL) were added into 100 μL riboflavin solution (0.2 mM in 0.1 M NaOH). After illuminating the mixture for 1 min, 150 μL NBT was introduced, and the absorption spectra were recorded for further analysis

Measurement of Lewis acidity by NMA fluorescence probe. Both MOFs and NMA solution (10 μM in acetonitrile) were first degassed with N_2 for 30 mins. Then, 10 mg MOFs were dispersed in 4 mL NMA solution by sonicating for 10 mins. The emission spectra were recorded using an excitation wavelength of 360 nm.

Monitoring of Lewis acid site-bound with superoxide species via EPR. The degassed MOFs were dispersed in riboflavin solution (1 mM in 0.1 M NaOH), and the resulting mixture was sonicated for 10 mins until the MOFs were well suspended. Then, the mixture was transferred into an EPR tube. After illumination for 5 mins, EPR spectra were recorded.

Evaluation of AChE-like activity of MOFs toward ATCh. For ATCh hydrolysis, 2 mg mL^{-1} MOFs (10 μL) and 1 mM ATCh (50 μL) were added into the 0.1 M HEPES buffer solution (pH 9.0, 500 μL) and incubated for 20 mins. The supernatant was obtained by centrifuging. Then, 4 mM DTNB (dissolved in dimethylsulfoxide (DMSO), 150 μL) was introduced into the supernatant (150 μL). After incubation for 3 mins, the absorption spectra were recorded for further analysis.

Kinetic assay of AChE-like activity. 2 mg mL^{-1} MOFs (30 μL) and different concertations of ACh (1, 5, 8, 10, 15, 20, 25, 30 mM) (250 μL) were added into 0.1 M HEPES buffer solution (pH 9.0, 0.5 mL). The supernatant was collected every 2 mins. Then, 1 U mL^{-1} ChOx (10 μL), 50 μg mL^{-1} HRP (10 μL), and TMB (dissolved in ethanol, 1 mM, 130 μL) were introduced into the supernatant (150 μL) for co-incubation for an additional 8 mins. The absorbance value at 652 nm was recorded using a microplate reader for further analysis. The Michaelis-Menten equation ($v=V_{max}[S]/(K_M + [S])$) was used to analyze the kinetics date of catalysts. The v is the initial velocity, V_{max} is the maximal reaction velocity, [S] is the concentration of the substrate, and K_M is the Michaelis−Menten constant.

As for ATCh, 2 mg mL^{-1} MOFs (50 μL) and different concertations of ATCh (1, 5, 8, 10, 15, 20, 25, 30 mM) (250 μL) were added into the 0.1 M HEPES buffer solution (pH 9.0, 0.5 mL). The supernatant was collected every 2 mins. Subsequently, 4 mM DTNB (dissolved in DMSO,150 μL) was introduced into the supernatant (150 μL) and incubated for 3 mins. The absorbance value at 460 nm was recorded using a microplate reader for further kinetic analysis.

Evaluation of OPH-like activity of MOFs. 2 mg mL^{-1} MOFs (10 μL) and 20 μL DENP (15 mM in 0.4 M N-ethylmorpholine) were added into the 270 μL H_2O solution and incubated for 15 mins. The absorption spectra were recorded by using the microplate reader for further analysis.

^{31}P-NMR spectra to track the hydrolysis products of DENP. 2 mg mL^{-1} MOFs (100 μL, in D_2O) and 20 μL DENP (15 mM in 0.4 M N-ethylmorpholine) were added into 1 mL D_2O and incubated for 30 mins. The supernatant was obtained and filtered through a 220 nm filter. The filtrates were analyzed using ^{31}P-NMR spectroscopy to monitor the product of reactions.

Kinetic assay of OPH-like activity. 2 mg mL^{-1} MOFs (10 μL) and 20 μL different concertations

of DENP (in 0.4 M N-ethylmorpholine) were added into the 270 μL H_2O solution. The absorbance value of the reaction solution (at 405 nm) was recorded by the microplate reader. Then, the Michaelis-Menten equation ($v=V_{max}[S]/(K_m + [S])$) was used to analyze the kinetics date of catalysts.

Evaluation of the stability of MOFs and AChE. 2 mg mL^{-1} MOFs and AChE (50 mU mL^{-1}) were treated at a high temperature (80 °C) for 1 h. Then, the activities of the treated MOFs and AChE were determined by using the above-mentioned method. Likewise, H_2O (50 μL), DMF (50 μL), and acetonitrile (MeCN, 50 μL) were also used to study the stability.

Evaluation of the recyclability of MOF-808-Al. The as-prepared MOF-808-Al first reacted with ACh, and the resulting absorption spectra were recorded. MOF-808-Al was then re-obtained via centrifuging (9500 xg, 3 min) and washed with H_2O. The re-obtained catalyst was subsequently used to react with DENP, and the same process was repeated several times.

Evaluation of the hydrolase-like activity of Al_2O_3. The evaluation methods of AChE and OPH-like activities are similar to the above-mentioned method, with the exception that Al_2O_3 nanomaterial (0.069 mg mL^{-1}), which is equivalent to the amount of Al in MOF-808-Al, was employed to replace the obtained MOFs.

DFT Calculation. DFT calculation is carried out by the Vienna Ab initio Simulation Package (VASP, version 5.4.4).[1] The elemental core and valence electrons were represented by the projector augmented wave (PAW) method and plane-wave basis functions with a cutoff energy of 450 eV. Generalized gradient approximation with the Perdew-Burke-Ernzerh of (GGA-PBE) exchange-correlation functional was employed in all the calculations.[2] Geometry optimizations were performed with the force convergency smaller than 0.05 eV/Å. The DFT-D3 empirical correction method was employed to describe van der Waals interactions.[3] Based on the MOF crystal structure, a cluster model was constructed, which contains more than 140 atoms, respectively. The terminal C atoms are saturated with H atoms. The terminal C-H bonds were optimized with all H atoms relaxed, while the other atoms fixed. A (1×1×1) k-point sampling with the Gamma-centered scheme was used for the Brillouin zone integration. Lattice parameter: a = b = c = 30 Å, α =90°, β = 90°, γ = 90°. The energy change of the reaction is calculated as equations 1-3:

Supplementary Equation (1) ACh + OH* → ACh* + OH*

$$\Delta E = E(ACh^* + OH^*) - E(ACh) - E(OH^*)$$

Supplementary Equation (2) ACh* + OH* → ACh-OH*

$$\Delta E = E(ACh^* - OH^*) - E(ACh + OH^*)$$

Supplementary Equation (3) ACh-OH* → $C_2H_3O_2$* + choline

$$\Delta E = E(choline) + E(C_2H_3O_2^*) - E(ACh^* - OH^*)”$$

Cell cultures. PC12 cells were cultured in Dulbecco's Modified Eagle's Medium (DMEM) medium (Gibco, China) containing 1% penicillin G sodium/streptomycin sulfate and 5% fetal bovine serum (FBS) (Gibco, China) at 37 °C with 5% CO_2 in a constant temperature incubator.

In vitro cytotoxicity assay. MTT assay was used to determine the cytotoxicity of MOFs. Briefly, PC12 cells were seeded into 96-well plates at a density of 10^4 cells per well (200 μL) and were cultured at 37 °C with 5% CO_2 for 24 h. Then MOFs in different concentrations were added before another incubation for 24 h. Subsequently, the medium was removed and only DMEM solution (100 μL) and 10 μL of MTT (5 mg mL^{-1}) were introduced. After incubating for 4 h, the media was removed and DMSO (150 μL) was added to each well. Absorbance values at 490 nm were determined with 630 nm as the reference. The cell viability was estimated according to the following equation: Cell viability (%) = ($A_{Treated}/A_{control}$) × 100%, where $A_{Treated}$ and $A_{Control}$ is the absorbance value with and without treatment of MOFs, respectively.

Mitochondrial membrane potential assay. PC12 cells (1 mL, 10^5 cells) were seeded into confocal dish for 24 h. After incubating cells with MOF-808-Al, DENP, and MOF-808-Al+DENP, 200 μL medium containing JC-1 dye (10 μg mL^{-1}) was introduced and incubated for 20 mins at 37 °C and 5% CO_2. The pre-treated cells were washed with a fresh medium and incubated in

a fresh culture medium. The corresponding fluorescence spectra were recorded and visualized using CLSM. JC-1 monomers were tested with an excitation wavelength of 488 nm and an emission wavelength of 530 nm. JC-1 aggregates were tested with an excitation wavelength of 530 nm and an emission wavelength of 595 nm.

Intracellular ROS detection by using DCFH-DA. PC12 cells (1 mL, 10^4 cells) were seeded into confocal dish for 24 h. After incubation with MOF-808-Al, DENP, and MOF-808-Al+DENP, PC12 cells were stained with a 2 μM DCFH-DA ROS probe for 30 mins and visualized using CLSM**.**

Detection of ACh by using MOF-808-Al-based biosensor. 2 mg mL^{-1} MOFs (10 μL) and different concentrations of ACh (80 μL) were added into the 0.1 M HEPES buffer solution (pH 9.0, 100 μL) and incubated for 30 mins. Then, 1 U mL^{-1} ChOx (10 μL), 50 μg mL^{-1} HRP (10 μL), and TMB (1 mM, 100 μL) were introduced and co-incubated for another 8 mins. The absorption spectra were recorded by the microplate reader for further analysis.

As for assay ACh concentration in intracellular, PC12 cells were seeded into 24-well plates at several densities of cells (10^4, 10^5, and 10^6) per well (200 μL), and were cultured at 37 °C with 5% CO_2 for 24 h. The medium was removed and the cells were washed thrice with PBS. The radioimmuno-precipitation assay (RIPA) buffer with 1 mM phenylmethylsulfonyl fluoride (PMSF) was added and incubated for 30 mins at 4 °C. The homogenate was centrifuged at 9500 xg at 4 °C and maintained for 10 mins. Then, 2 mg mL^{-1} MOF-808-Al (10 μL) and 0.1 M HEPES buffer solution (pH 9.0, 100 μL) were added into the obtained solution and incubated for 30 mins. Then, 1 U mL^{-1} ChOx (10 μL), 50 μg mL^{-1} HRP (10 μL), and TMB (1 mM, 130 μL) were further introduced and co-incubated for another 8 mins. The absorption spectra were recorded by the microplate reader.

Biochemical analysis. To evaluate the toxic effects induced by DENP, the level of various biomarkers in mice, including AChE, SOD, ALP, GSH, and MDA, was detected. The AChE activity in the serum of mice was determined using the above-mentioned method. For the expression of ALP and SOD, GSH, and MDA analysis, the corresponding reagent kits were employed, respectively.

Statistical analysis: All data in this study were recorded as mean ± standard deviation (s.d.). Student's t-tests were performed to analyze the statistical results. The data were analyzed by using the Statistical Package for the Social Sciences (SPSS, version 26).

Figures and Tables

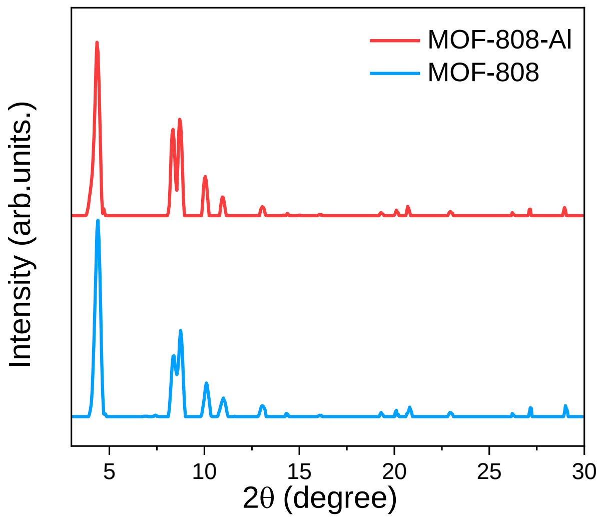

Supplementary Fig. 1. XRD patterns of MOFs.

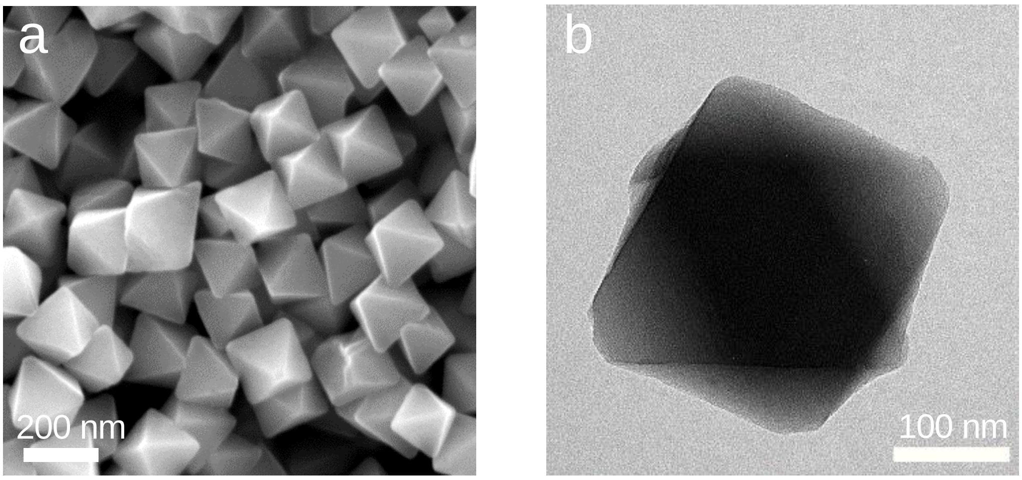

Supplementary Fig. 2. (a) SEM image of MOF-808. (b) TEM image of MOF-808-Al. The experiments were repeated three times with similar results.

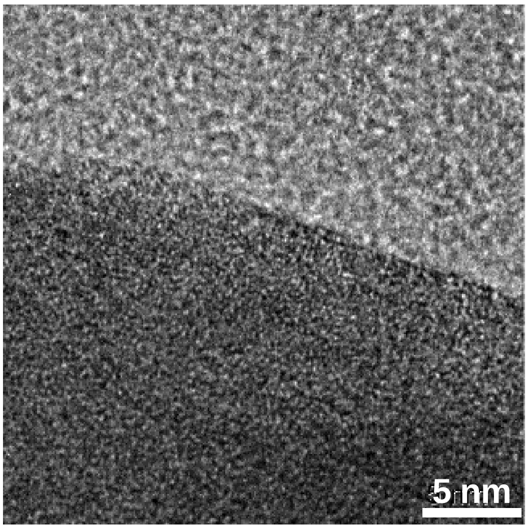

Supplementary Fig. 3. High-resolution TEM image of MOF-808-Al. The experiment was repeated three times with similar results.

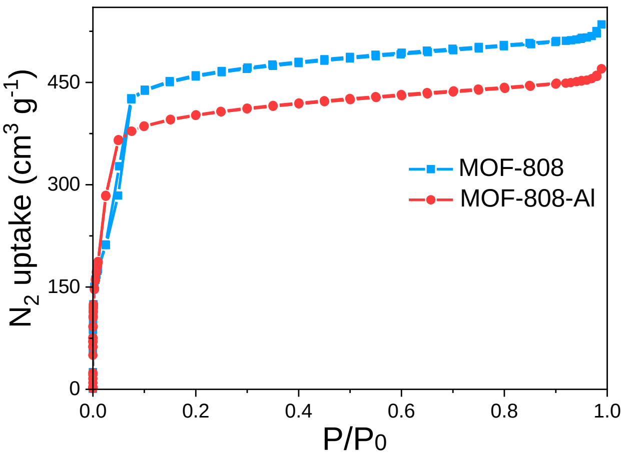

Supplementary Fig. 4. N_2 sorption isotherms for MOFs at 77 K.

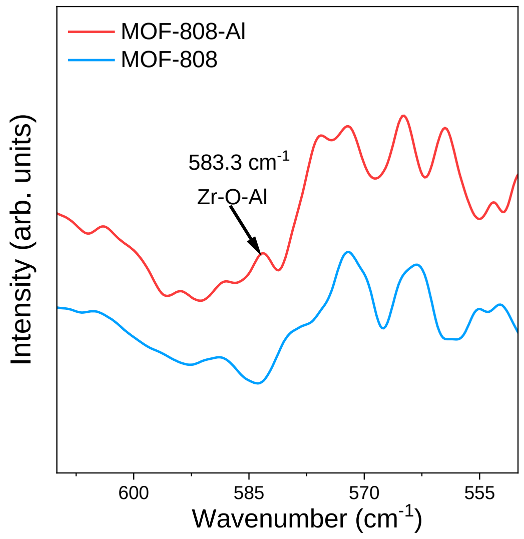

Supplementary Fig. 5. FTIR spectra of MOF-808 and MOF-808-Al.

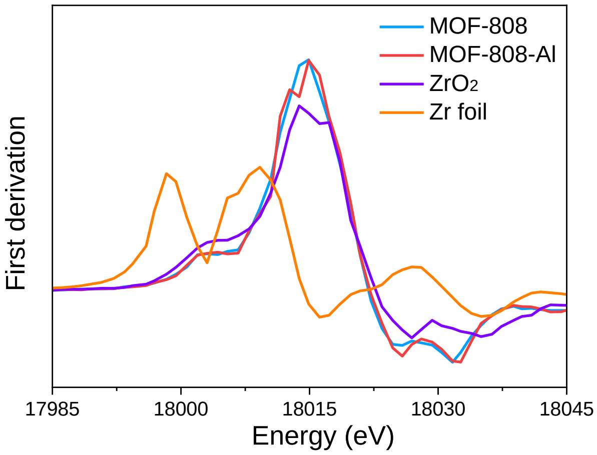

Supplementary Fig. 6. First derivation of XANES curves of MOFs and references.

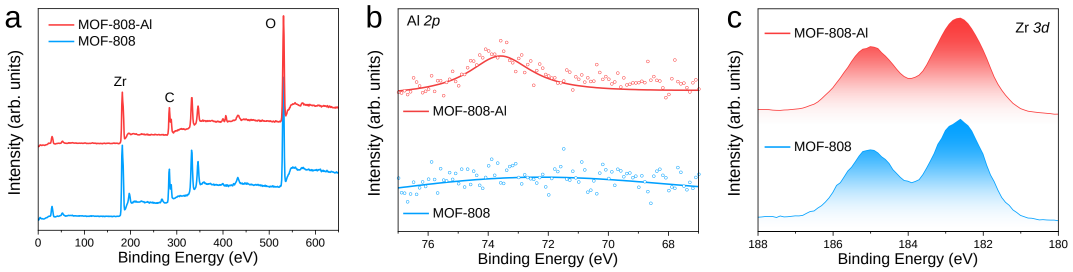

Supplementary Fig. 7. (a) XPS survey scan spectra of MOFs. (b) Al 2p and (c) Zr 3d XPS spectra of MOFs.

Compared with the MOF-808, the signal at 73.6 eV of MOF-808-Al is attributed to Al^{3+} (Supplementary **Fig. 7**b). After modification of Al species, the oxidation state of Zr remains unaltered at +4, which is consistent with the XANES results (Supplementary **Fig. 7**c).

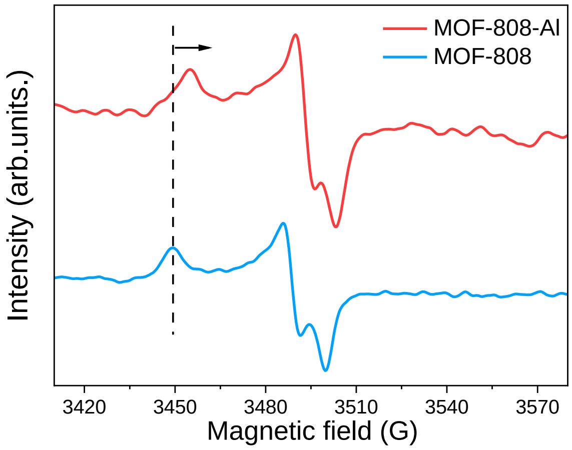

Supplementary Fig. 8. EPR spectra of MOFs reacting with $O_2^{\bullet-}$.

For the MOF-808 reaction system, these characteristic signal peaks are attributed to the Zr-$O_2^{\bullet-}$ species, verifying the interaction between Lewis acid sites and electron-rich $O_2^{\bullet-}$. In comparison, the signal peak of the MOF-808-Al system exhibits a redshift, which may be attributed to the presence of Al^{3+} Lewis sites.

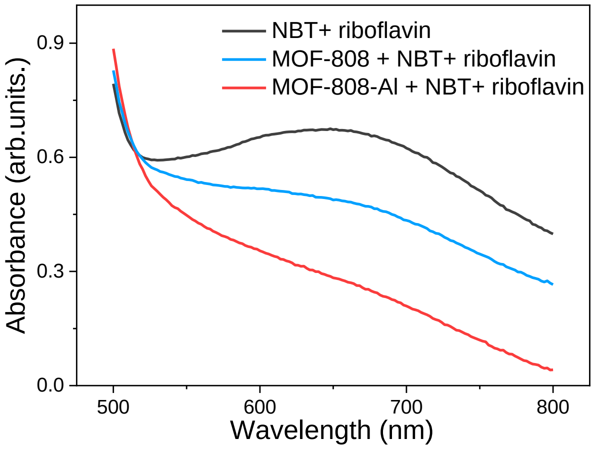

Supplementary Fig. 9. Absorption spectra of different reaction systems, including NBT + riboflavin, MOF-808 + NBT + riboflavin, and MOF-808-Al + NBT + riboflavin.

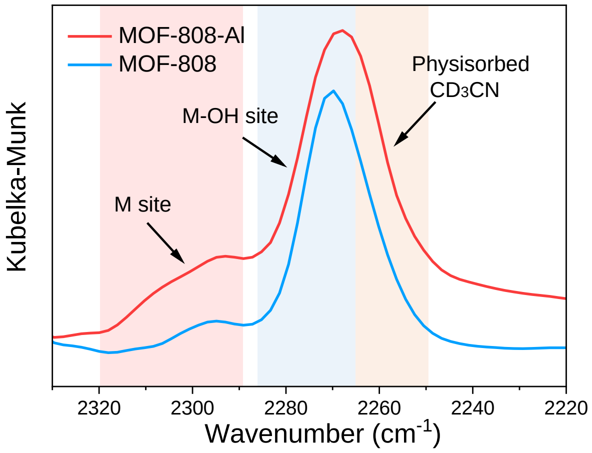

Supplementary Fig. 10. FTIR spectra of CD_3CN chemisorption on MOFs. The pink means M site, blue means M-OH site, and organe means physisorbed CD_3CN.

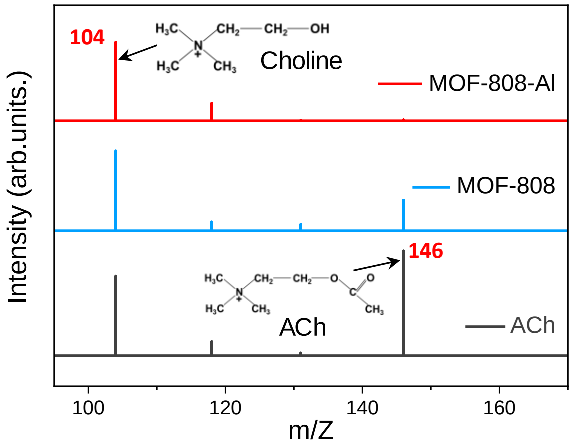

Supplementary Fig. 11. Mass spectra of ACh before and after catalyzed by MOFs.

After reaction with MOFs, the relative signal intensity of choline increases significantly, indicating that obtained MOFs can perform the function of AChE to hydrolyze ACh into choline. Besides, the relative content of choline in the MOF-808-Al-involved system is higher than that in the MOF-808 system, corresponding to its better hydrolytic activity.

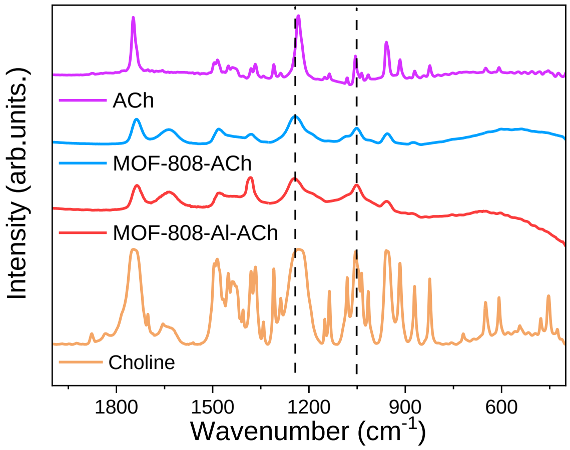

Supplementary Fig. 12. FTIR spectra of ACh, choline, and the supernatant of MOF-ACh reaction systems.

As can be seen in Supplementary **Fig. 12**, the peaks around 1247 cm^{-1} and 1050 cm^{-1}, assigned to the -OH bond and C-O bond of primary alcohol vibrations, are observed in both reaction systems, demonstrating the generation of choline.

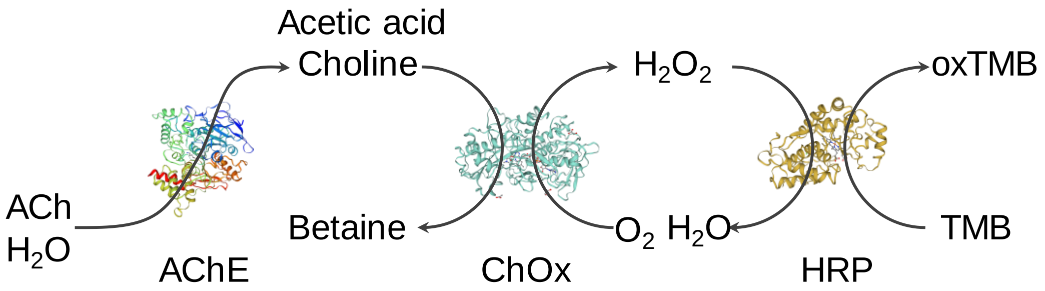

Supplementary Fig. 13. Schematic illustration of the AChE-ChOx-HRP cascade reactions.

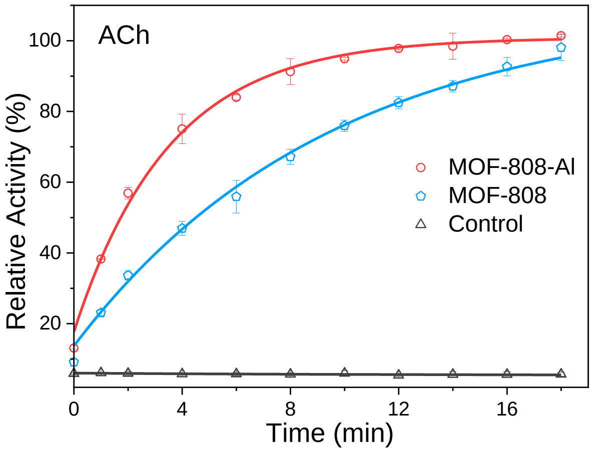

Supplementary Fig. 14. Relative hydrolytic activity of MOFs for ACh. Error bars mean ± s.d. calculated from three independent measurements.

Relative activity (%) = (A_{Final}/A_i) × 100%. A_{Final} is the final absorbance value of the MOF-808-Al-catalyzed reaction system. A_i is the absorbance value of different reaction systems at various times.

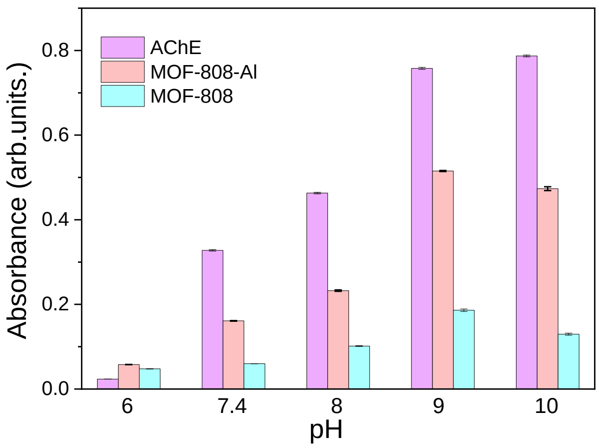

Supplementary Fig. 15. Absorbance values (652 nm) of ACh hydrolysis reaction in different pH buffer solutions. Error bars mean ± s.d. calculated from three independent measurements.

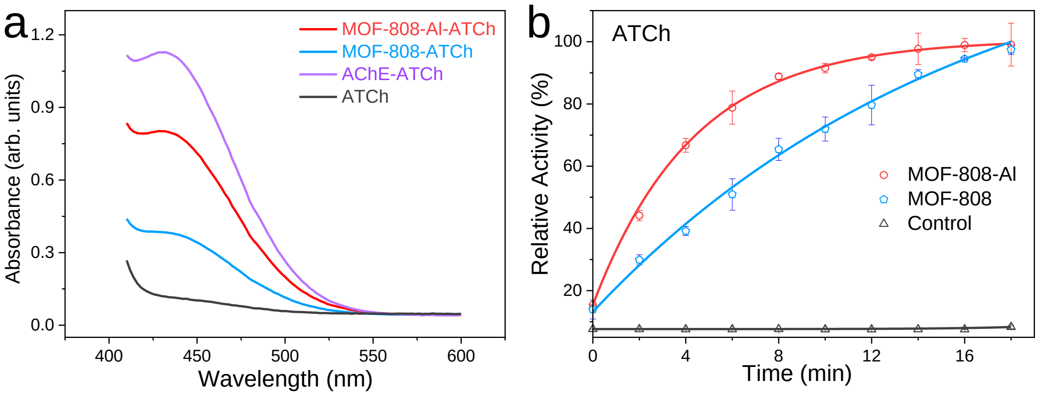

Supplementary Fig. 16. (a) Absorption spectra of ATCh hydrolytic reaction catalyzed by AChE, MOF-808, and MOF-808-Al, respectively. (b) The relative hydrolytic activity of MOFs for ATCh. Error bars mean ± s.d. calculated from three independent measurements.

As shown in Supplementary **Fig. 16**a, both MOFs exhibit AChE-like activity to hydrolyze ATCh into thiocholine, which can further react with DTNB to form a yellow product with maximum absorption at 405 nm. Compared with the MOF-808 system, the MOF-808-Al system exhibits a higher absorbance value at 405 nm, implying its superior hydrolytic performance. Moreover, the MOF-808-Al system displays a faster hydrolytic rate than the MOF-808 system (Supplementary **Fig. 16**b).

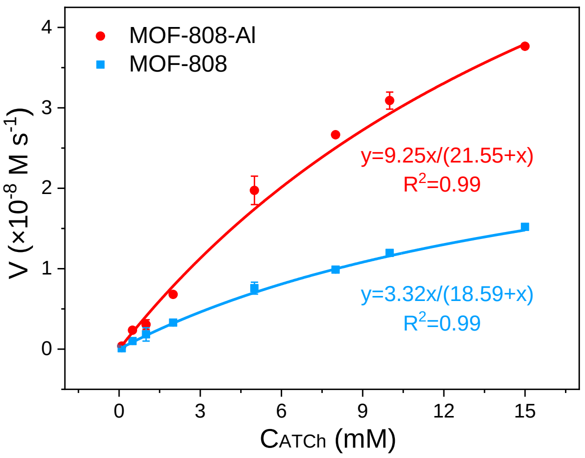

Supplementary Fig. 17. The kinetic curves of MOF-808 and MOF-808-Al for hydrolysis of ATCh. Error bars mean ± s.d. calculated from three independent measurements.

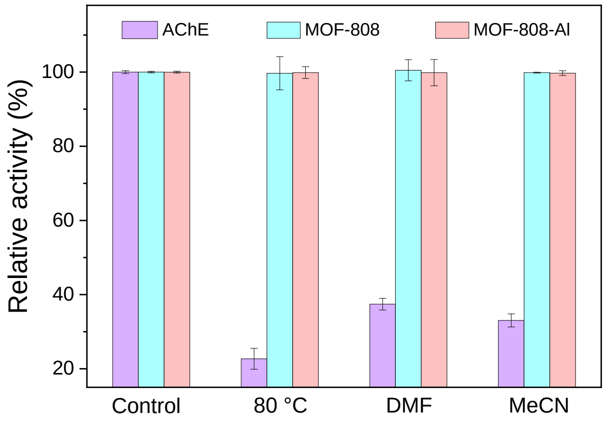

Supplementary Fig. 18. Relative activity of AChE, MOF-808, and MOF-808-Al before and after treatment with high temperature and small organic molecules. Error bars mean ± s.d. calculated from three independent measurements.

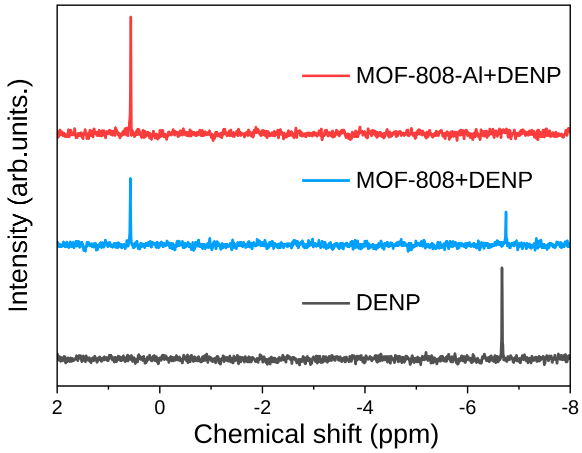

Supplementary Fig. 19. ^{31}P-NMR spectra of DENP hydrolysis reaction catalyzed by MOF-808 and MOF-808-Al.

For the MOF-808 system, the characteristic peak of DENP (around -6.8 ppm) decreases, while a peak appears at around 1.0 ppm, indicating the conversion of DENP to diethylene phosphate anion. Notably, the characteristic peak of DENP is not detected in the MOF-808-Al system. These results demonstrate that the hydrolytic activity of the MOF-808-Al is higher than that of the MOF-808.

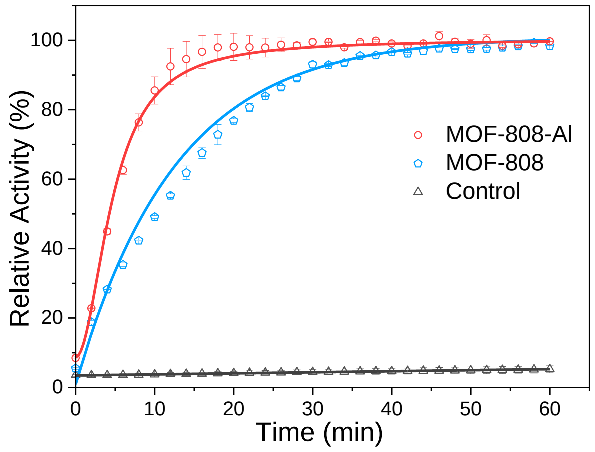

Supplementary Fig. 20. Relative hydrolytic activity of MOFs toward DENP. Error bars mean ± s.d. calculated from three independent measurements.

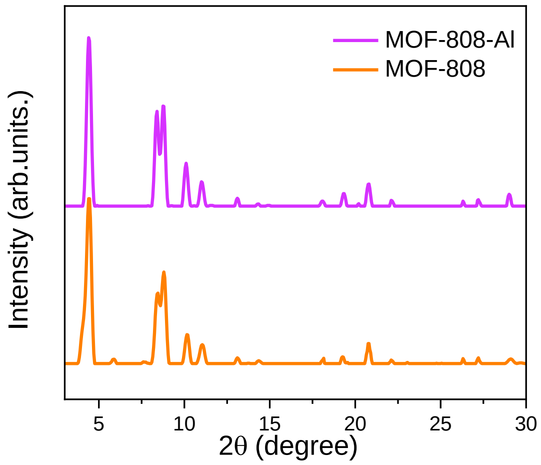

Supplementary Fig. 21. XRD patterns of MOFs after treatment with DENP.

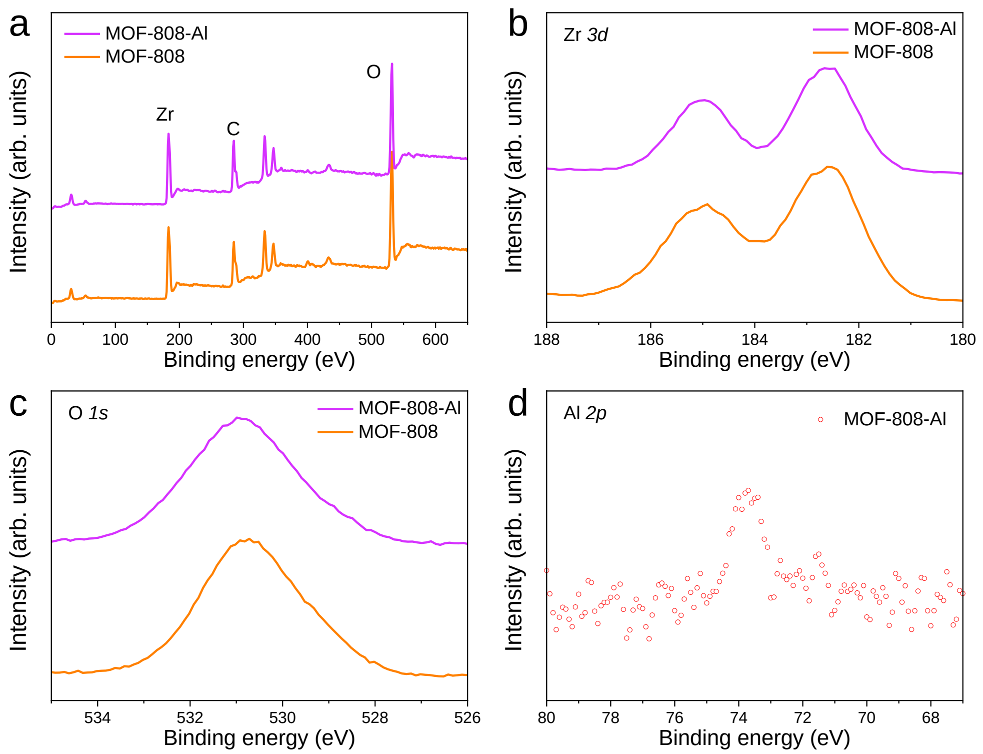

Supplementary Fig. 22. (a) XPS survey scan spectra of MOFs after treatment with DENP. (b) Zr *3d* and (c) O *1s* spectra of MOFs after treatment with DENP. (d) Al *2p* spectrum of DENP-treated MOF-808-Al.

After treatment with DENP, the characteristic peaks of various elements over MOFs exhibit no perceptible change compared to that of pristine MOFs.

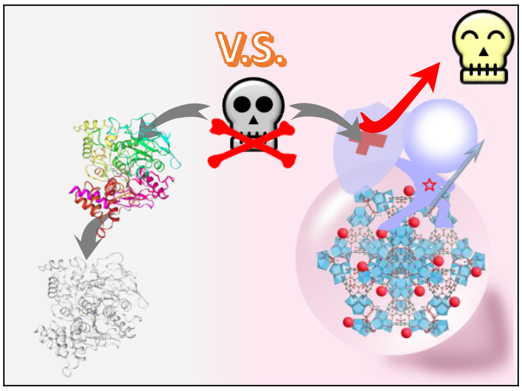

AChE MOF-808-Al

Supplementary Fig. 23. Schematic illustration of anti-poisoning of AChE and MOF-808-Al toward DENP.

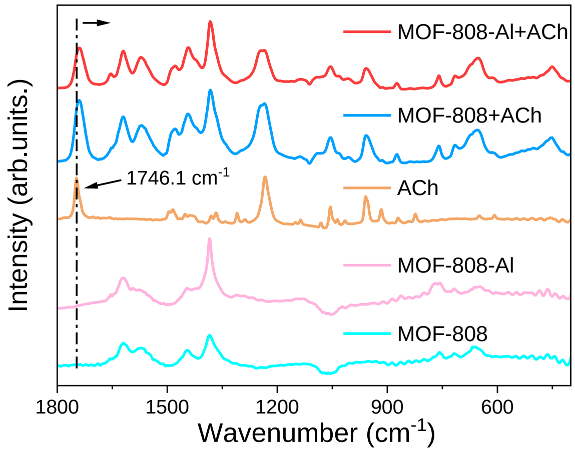

Supplementary Fig. 24. FTIR spectra of MOFs, ACh, MOF-808 + ACh, and MOF-808-Al + ACh.

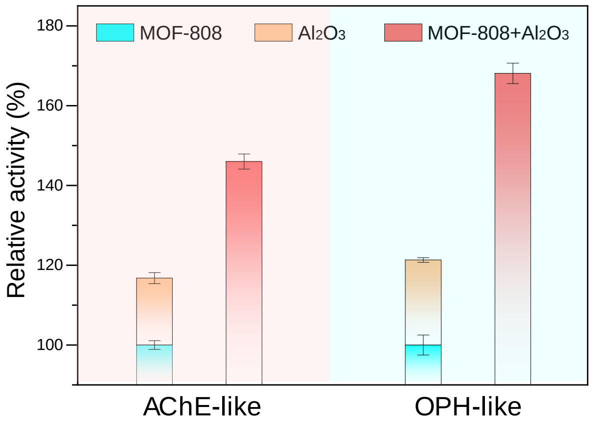

Supplementary Fig. 25. The relative hydrolytic activity of Al_2O_3, MOF-808, and Al_2O_3 + MOF-808 integrated system. Error bars mean ± s.d. calculated from three independent measurements. The pink indicates AChE-like (left), blue indicates OPH-like (right).

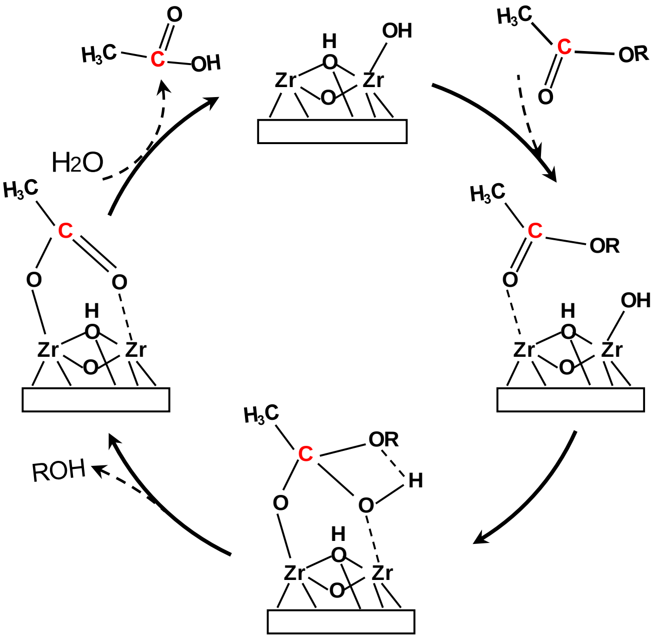

Supplementary Fig. 26. Schematic illustration of the hydrolysis process of ACh on Zr-MOF.

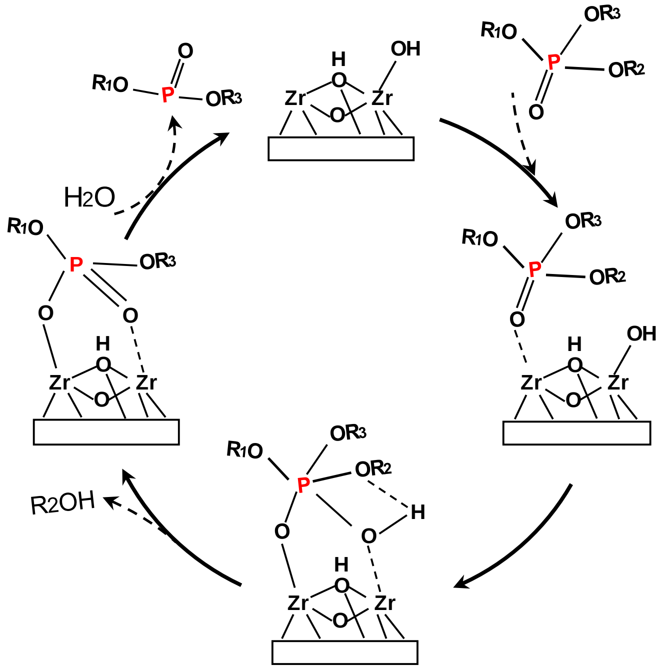

Supplementary Fig. 27. Schematic illustration of the hydrolysis process of DENP on Zr-MOF.

a b

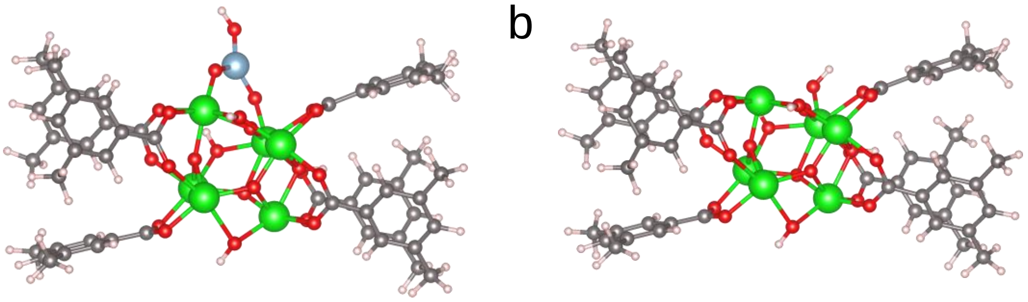

Supplementary Fig. 28. Schematic illustration of the structure of (a) MOF-808-Al and (b) MOF-808.

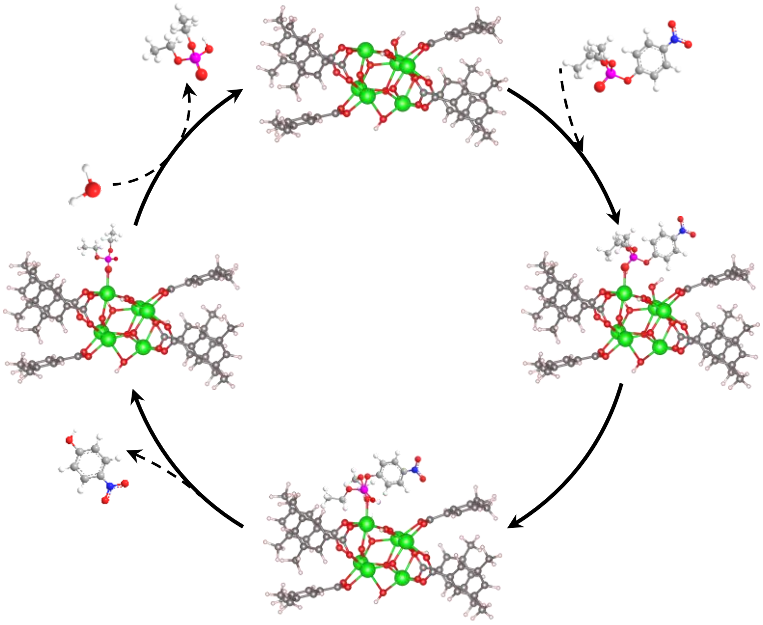

Supplementary Fig. 29. Proposed reaction process on MOF-808 for hydrolysis of ACh.

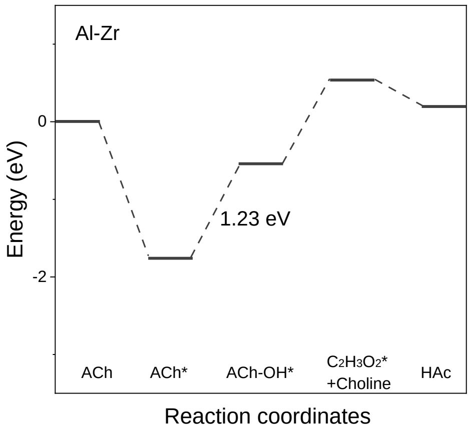

Supplementary Fig. 30. The energy diagram of the reaction process on Al-Zr sites in MOF-808-Al for hydrolysis ACh, where Al acts as a binding site, and Zr-OH acts as a nucleophilic attack group.

Transition state search indicates that the attack of Zr-OH* is controlled by thermodynamics.

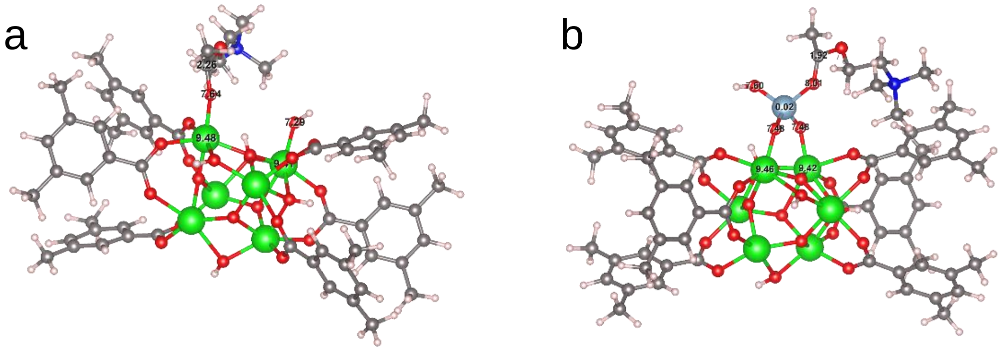

Supplementary Fig. 31. The valence electrons of (a) MOF-808 and (b) MOF-808-Al after binding with ACh.

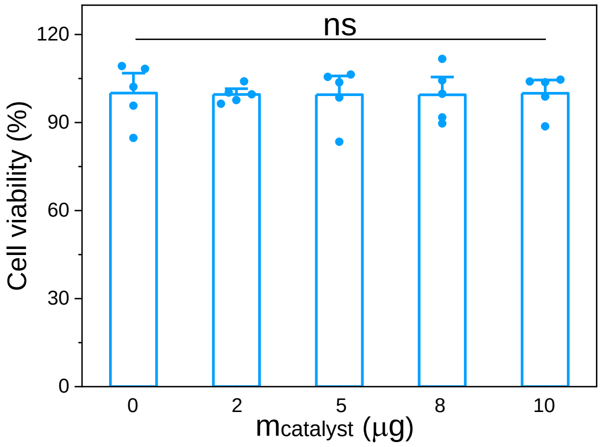

Supplementary Fig. 32. Cell viability after treatment with different contents of MOF-808. Data are represented as mean ± s.d. (n = 5 independent experiments), ns represents no statistical difference.

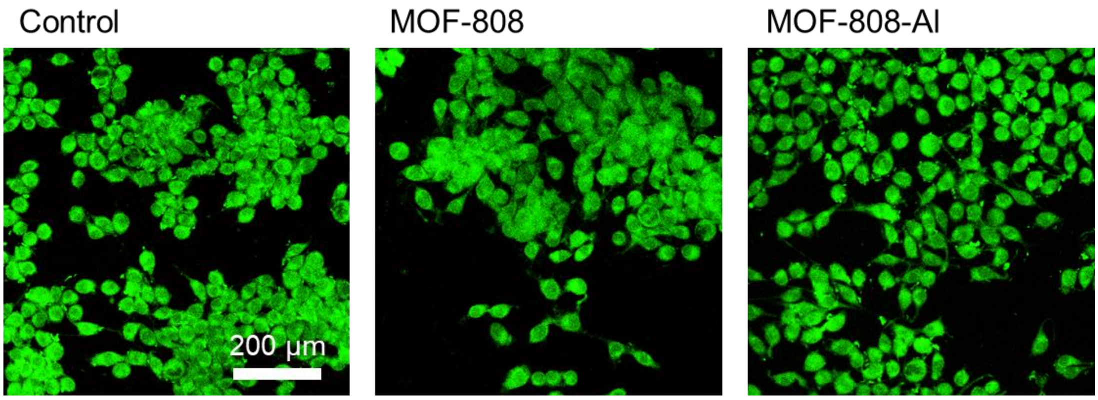

Supplementary Fig. 33. CLSM images of Calcein-AM/PI-stained cells treated with varying MOFs.

After treatment with MOF-808 and MOF-808-Al, no observed red fluorescence indicates the good biosafety of both MOFs.

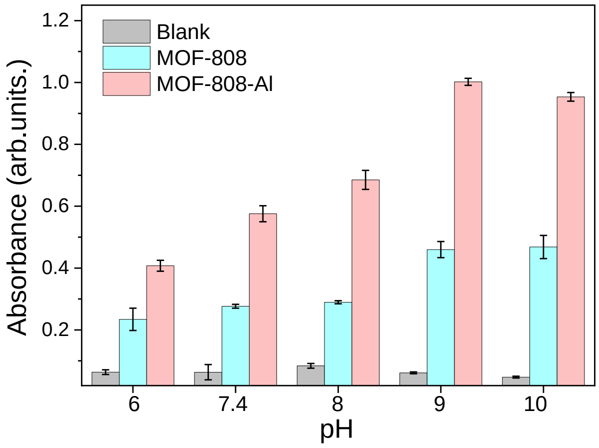

Supplementary Fig. 34. Absorbance values (at 652 nm) of DENP hydrolysis reaction under different pH conditions. Error bars mean ± s.d. calculated from three independent measurements.

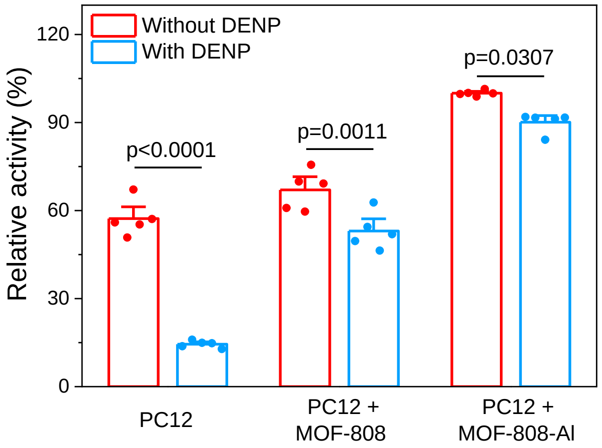

Supplementary Fig. 35. The relative activity of AChE in PC12 cells before and after treatment with DENP in the presence of MOF-808 and MOF-808-Al. Data are represented as mean ± s.d. (n = 5 independent experiments).

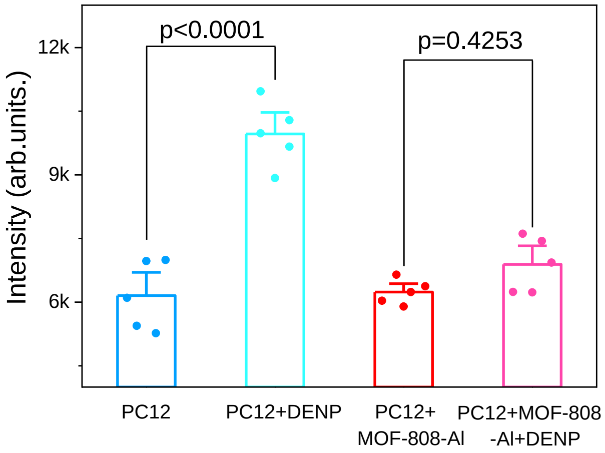

Supplementary Fig. 36. Fluorescence intensity of JC-1 (E_m: 530 nm) in different systems, including PC12, PC12 + DENP, PC12 + MOF-808-Al, and PC12 + MOF-808-Al + DENP. Data are represented as mean ± s.d. (n = 5 independent experiments).

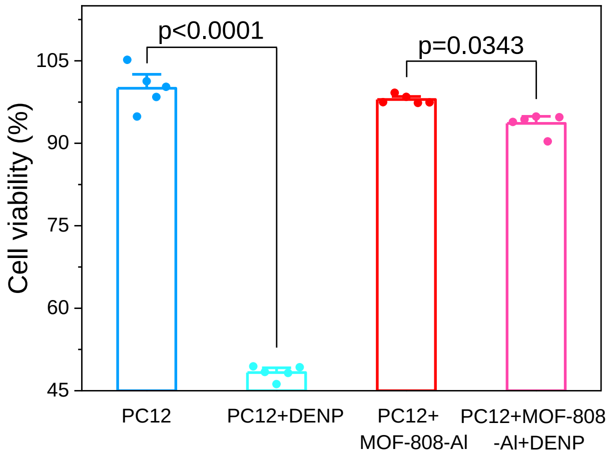

Supplementary Fig. 37. Cell viability of PC12 cell before and after treatment with DENP in the absence and presence of MOF-808-Al. Data are represented as mean ± s.d. (n = 5 independent experiments).

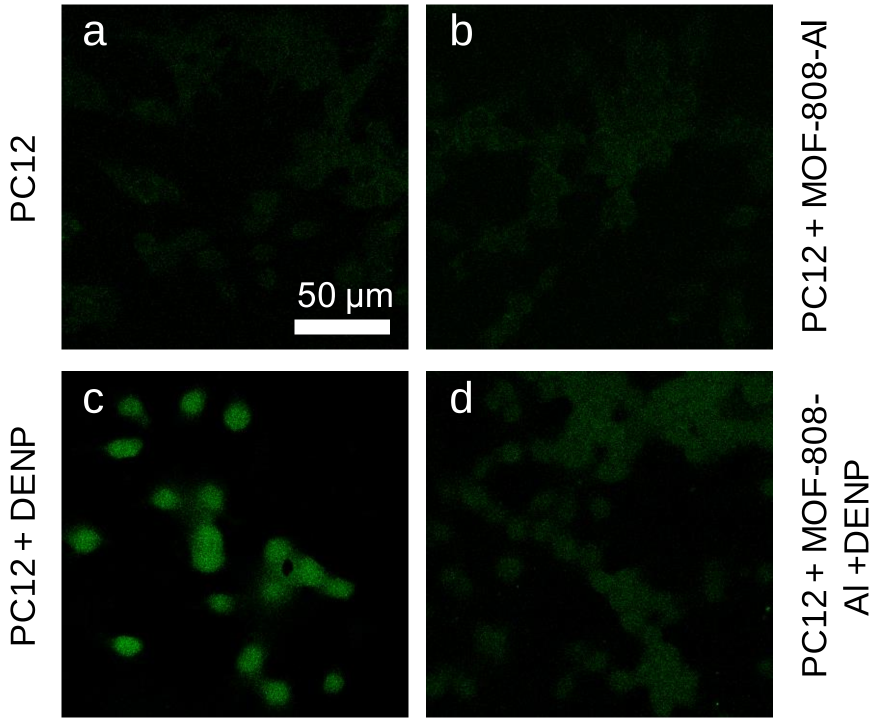

Supplementary Fig. 38. CLSM images of DCFH-DA-stained PC12 cells after treatment with DENP, MOF-808-Al, and MOF-808-Al + DENP.

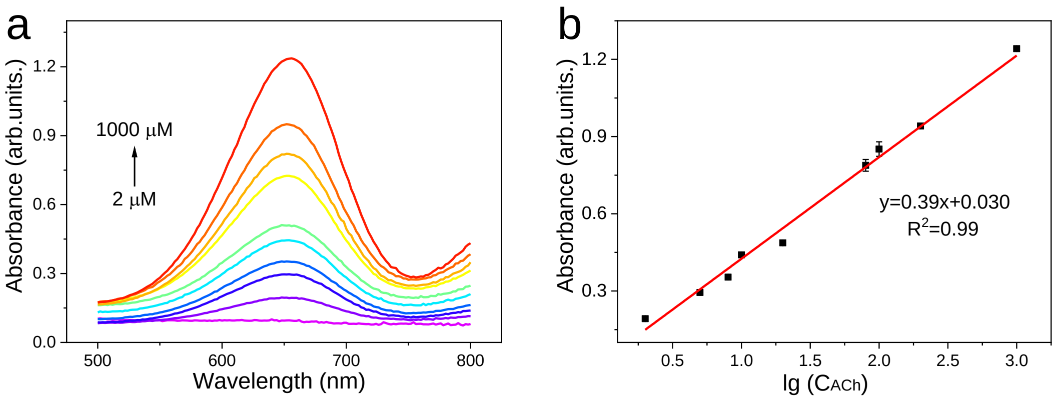

Supplementary Fig. 39. (a) Absorption spectra for the detection of different concentrations of ACh by using the MOF-808-Al-based biosensor, (b) and corresponding linear equation between absorbance values (at 652 nm) and the logarithm of ACh concentrations. Error bars mean ± s.d. calculated from three independent measurements.

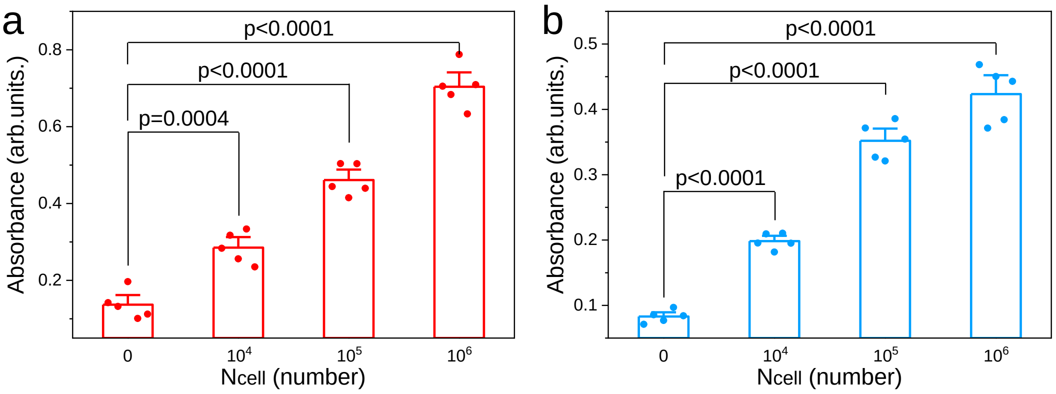

Supplementary Fig. 40. Absorbance values (at 652 nm) for the detection of the ACh concentrations in various numbers of PC12 cells by using the (a) MOF-808-Al and (b) MOF-808 biosensors. Data are represented as mean ± s.d. (n = 5 independent experiments).

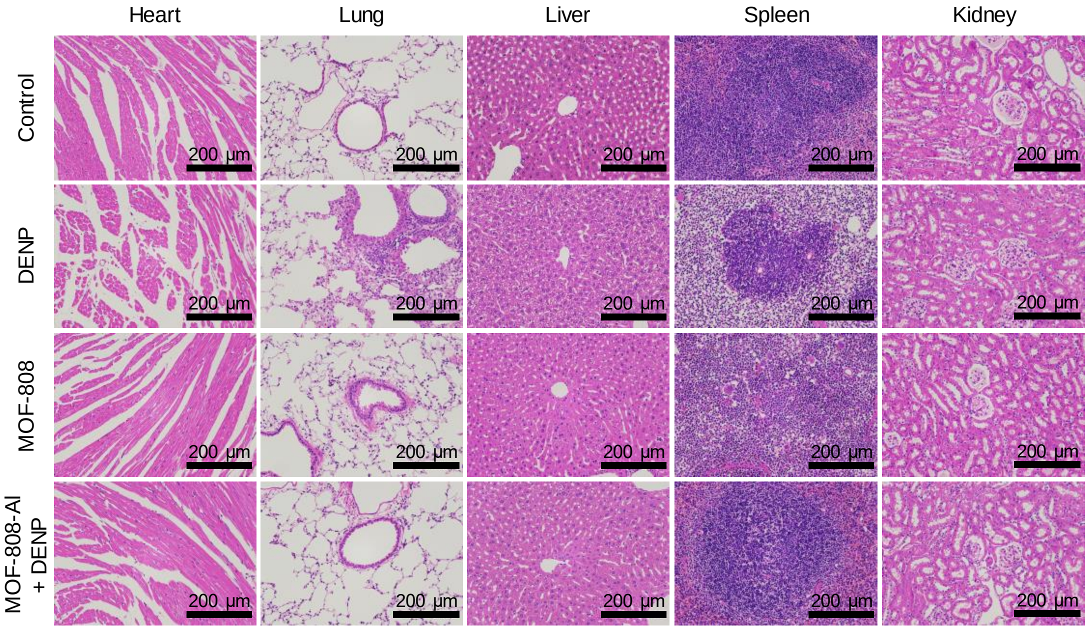

Supplementary Fig. 41. H&E staining of heart, lung, liver, spleen, and kidney of mice after treatment with DENP, MOF-808-Al, and MOF-808-Al + DENP.

Supplementary Table 1. The V_{max}, $K_{M,}$ and catalytic constants of both MOFs toward ACh.

Catalysts	V_{max} (10^{-8} M s^{-1})	K_M (mM)	K_{cat} (min^{-1})	K_{cat}/K_M (M^{-1} min^{-1})
MOF-808	4.39	28.69	0.038	1.32
MOF-808-Al	8.35	19.66	0.072	3.66

K_{cat} = V_{max}/[E], [E] is the concentration of the catalyst.

Supplementary Table 2. The V_{max}, $K_{M,}$ and catalytic constants of both MOFs toward ATCh.

Catalysts	V_{max} (10^{-8} M s^{-1})	K_M (mM)	K_{cat} (min^{-1})	K_{cat}/K_M (M^{-1} min^{-1})
MOF-808	3.32	18.59	0.029	1.56
MOF-808-Al	9.25	21.55	0.078	3.62

Supplementary Table 3. The V_{max}, K_M, and catalytic constants of both MOFs toward DENP.

Catalysts	V_{max} (10^{-8} M s^{-1})	K_M (mM)	K_{cat} (min^{-1})	K_{cat}/K_M (M^{-1} min^{-1})
MOF-808	28.40	3.88	0.73	188.14
MOF-808-Al	57.98	0.78	1.50	1923.08

Supplementary Table 4. The detection of ACh using the MOF-808-Al and DENP-treated MOF-808-Al.

Spiked Concentration (μM)	Detected concentration (μM)		RSD (%, n=4)
	MOF-808-Al	DENP-treated MOF-808-Al	
5	4.91	4.95	1.08
10	10.03	9.78	0.17
100	99.51	101.38	1.86
200	203.7	196.76	1.31
500	502.40	505.52	2.11

Supplementary Reference

1. Botana, AS. Norman MR. Electronic structure and magnetism of transition metal dihalides: bulk to monolayer. *Phy. Rev. Mater.* **3**, 044001 (2019).
2. Wang, J. *et al.* Porphyrin Conjugated Polymer with Periodic Type II-Like Heterojunctions and Single-Atom Catalytic Sites for Broadband-Responsive Hydrogen Evolution. *Adv. Funct. Mater.* **31**, 2009819 (2021).
3. Grimme, S. Antony, J. Ehrlich, S. Krieg, H. A consistent and accurate ab initio parametrization of density functional dispersion correction (DFT-D) for the 94 elements H-Pu. *J. Chem. Phys.* **132**, 154104 (2021).
