# Supplementary material for: Biomimetic single Al-OH site with high acetylcholinesterase-like activity and self-defense ability for neuroprotection
Source: Nat Commun. 2023 Sep 28;14:6064. doi: 10.1038/s41467-023-41765-x (PMC10539540; doi:10.1038/s41467-023-41765-x)
Supplement: Supplementary file 2 — Reporting Summary [file 41467_2023_41765_MOESM2_ESM.pdf]

nature portfolio

Corresponding author(s): Prof. Shaojun Guo

Last updated by author(s): Sep 16, 2023

Reporting Summary

Nature Portfolio wishes to improve the reproducibility of the work that we publish. This form provides structure for consistency and transparency in reporting. For further information on Nature Portfolio policies, see our Editorial Policies and the Editorial Policy Checklist.

Statistics

For all statistical analyses, confirm that the following items are present in the figure legend, table legend, main text, or Methods section.

n/a	Confirmed	
☐	☒	The exact sample size (*n*) for each experimental group/condition, given as a discrete number and unit of measurement
☐	☒	A statement on whether measurements were taken from distinct samples or whether the same sample was measured repeatedly
☐	☒	The statistical test(s) used AND whether they are one- or two-sided<br>*Only common tests should be described solely by name; describe more complex techniques in the Methods section.*
☒	☐	A description of all covariates tested
☒	☐	A description of any assumptions or corrections, such as tests of normality and adjustment for multiple comparisons
☐	☒	A full description of the statistical parameters including central tendency (e.g. means) or other basic estimates (e.g. regression coefficient) AND variation (e.g. standard deviation) or associated estimates of uncertainty (e.g. confidence intervals)
☐	☒	For null hypothesis testing, the test statistic (e.g. *F*, *t*, *r*) with confidence intervals, effect sizes, degrees of freedom and *P* value noted<br>*Give P values as exact values whenever suitable.*
☒	☐	For Bayesian analysis, information on the choice of priors and Markov chain Monte Carlo settings
☐	☐	For hierarchical and complex designs, identification of the appropriate level for tests and full reporting of outcomes
☒	☐	Estimates of effect sizes (e.g. Cohen's *d*, Pearson's *r*), indicating how they were calculated

Our web collection on statistics for biologists contains articles on many of the points above.

Software and code

Policy information about availability of computer code

Data collection	Transmission electron microscopy (TEM) studies were performed by using a FEI Titan G2 60-300 (super-x). Scanning electron microscopy (SEM) images were obtained from the Gemini SEM 300 (ZEISS). X-ray absorption spectroscopy (XAS)experiments on a Zr K-edge were obtained from 1W1B station in Beijing Synchrotron Radiation Facility (BSRF). Inductively coupled plasma optical emission (ICP-OES) was utilized an Agilent 8800 spectrometry . Nitrogen sorption measurements were conducted using a fully automatic specific surface and aperture analyzer (Quantachrome, USA). Powder X-ray diffraction (XRD) patterns were carried out using a Tensor 27. X-ray photoelectron spectroscopy (XPS) measurements were used by a VG Multilab 2000 (Thermo Fisher, USA). Electron paramagnetic resonance (EPR) measurements were obtained by an EMXmicro-6/1 (Bruker, Germany). In-situ ATR-FTIR analysis was performed using a Nicolet iS50 FTIR spectrometer (Thermo). Confocal laser scanning microscopy (CLSM) experiment utilized a Leica TCS SP8 microscope. All the absorbance and fluorescence spectra were obtained from a multimode reader (Tecan Spark, Switzerland).
Data analysis	Athena and Artemis software were used to analyze the XAS data. DFT calculation is carried out by the Vienna Ab initio Simulation Package (version 5.4.4). Statistical analysis was used a Statistical Package for the Social Sciences (version 26).

For manuscripts utilizing custom algorithms or software that are central to the research but not yet described in published literature, software must be made available to editors and reviewers. We strongly encourage code deposition in a community repository (e.g. GitHub). See the Nature Portfolio guidelines for submitting code & software for further information.

Data

Policy information about availability of data

All manuscripts must include a data availability statement. This statement should provide the following information, where applicable:

- Accession codes, unique identifiers, or web links for publicly available datasets
- A description of any restrictions on data availability
- For clinical datasets or third party data, please ensure that the statement adheres to our policy

The data supporting the findings of this study are available within the article and its Supplementary Information files. All other data are available from the corresponding author(s) upon request.

Research involving human participants, their data, or biological material

Policy information about studies with human participants or human data. See also policy information about sex, gender (identity/presentation), and sexual orientation and race, ethnicity and racism.

Reporting on sex and gender	Not applicable
Reporting on race, ethnicity, or other socially relevant groupings	Not applicable
Population characteristics	Not applicable
Recruitment	Not applicable
Ethics oversight	Not applicable

Note that full information on the approval of the study protocol must also be provided in the manuscript.

Field-specific reporting

Please select the one below that is the best fit for your research. If you are not sure, read the appropriate sections before making your selection.

[x] Life sciences [ ] Behavioural & social sciences [ ] Ecological, evolutionary & environmental sciences

For a reference copy of the document with all sections, see nature.com/documents/nr-reporting-summary-flat.pdf

Life sciences study design

All studies must disclose on these points even when the disclosure is negative.

Sample size	Sample size choice was based on previous studies (ref. Huo, M., Tang, Z., Wang, L. et al, 2021. https://doi.org/10.1038/s41467-022-35503-y; Xu, Y., Li, C., Lu, S. et al. https://doi.org/10.1038/s41467-022-29572-2), not predetermined by a statistical method. Sample sizes were indicated in the legend of each Figure and Supplementary Figure.
Data exclusions	No data was excluded.
Replication	We confirm all attempts at replication were successful. The experimental findings were all replicated at least 3 times independently.
Randomization	All samples were randomly allocated into experimental groups.
Blinding	Investigators were not blinded for nanomaterial synthesis. For in vivo experiments, the investigators were blinded to group allocation during data collection and analysis.

Reporting for specific materials, systems and methods

We require information from authors about some types of materials, experimental systems and methods used in many studies. Here, indicate whether each material, system or method listed is relevant to your study. If you are not sure if a list item applies to your research, read the appropriate section before selecting a response.

Materials & experimental systems

n/a	Involved in the study	
x		Antibodies
	x	Eukaryotic cell lines
x		Palaeontology and archaeology
	x	Animals and other organisms
x		Clinical data
x		Dual use research of concern
x		Plants

Methods

n/a	Involved in the study	
x		ChIP-seq
x		Flow cytometry
x		MRI-based neuroimaging

Eukaryotic cell lines

Policy information about cell lines and Sex and Gender in Research

Cell line source(s)	PC12cells (mouse, ATCC CRL-1721.1)
Authentication	Cell line has not been authenticated.
Mycoplasma contamination	Mycoplasma test was negative.
Commonly misidentified lines (See ICLAC register)	No commonly misidentified cell lines was used.

Animals and other research organisms

Policy information about studies involving animals; ARRIVE guidelines recommended for reporting animal research, and Sex and Gender in Research

Laboratory animals	The healthy C57BL/6 mice (four weeks) were purchased from Hubei Provincial Center for Disease Control and Prevention.
Wild animals	No wild animals were used in this study.
Reporting on sex	We included both male and female mice in our study. We did not find any sex differences in any of our tests, therefore we combined male and female mice in all final statistical analyses.
Field-collected samples	No field-collected samples were used in this study.
Ethics oversight	The in vivo animal experiments were performed according to the guidelines of the National Institutes of Health Guide for the Care and Use of Laboratory Animals and approved by the Ethics Committee of Medical College, Wuhan University of Science and Technology (2023082).

Note that full information on the approval of the study protocol must also be provided in the manuscript.
